# Glycan targeting can recruit anti-tumor immunologic functions by lymphatic endothelium

**DOI:** 10.1016/j.neo.2026.101340

**Published:** 2026-07-30

**Authors:** Aditya Dash, Scott C. Johns, Chace Moleta, Elina I. Zúñiga, Mark M. Fuster

**Affiliations:** a VA San Diego Healthcare System, San Diego, USA; b Department of Molecular Biology, School of Biological Sciences, University of Californa, San Diego, USA; c Division of Pulmonary, Critical Care, and Sleep Medicine, University of California, San Diego, USA; d Veterans Medical Research Foundation, San Diego, CA, USA

**Keywords:** T cells, Lymphatic, Immunity, Cytotoxic t-lymphocyte, Cancer

## Abstract

During carcinoma progression, CD8+ T cells mediate tumor-cytolytic functions following tumor-antigen sensitization, typically by antigen cross-presenting dendritic cells. Lymphatic endothelial cells (LECs) are not typically endowed with such immune-effector capacity, and often promote tolerance. Inspired by insights from dendritic-cell glycan targeting, we examined how antigen-pulsed lymph node LECs genetically deficient in *Ndst1,* expressing under-sulfated heparan sulfate, might affect antigen-sensitized CD8+ T cell responses. Mutation significantly augmented responses to Ova-peptide pulsed LECs, including marked Nur77 activation along with more modest IFN-γ induction in co-cultured Ova-responsive CD8+ T cells, and increased antigen presentation relative to wildtype LECs (without marked differences in MHC-I, CD86 or PD-L1 immune checkpoint ligands). Tumor draining lymph nodes of lymphatic targeted tumor-bearing *Ndst1*f/f *Prox1Cre+* mutant mice showed reduced metastatic loads accompanied by augmented CD8+ T cell densities compared to *Ndst1*f/f *Prox1Cre*- controls. These findings provide mechanistic insight on harnessing glycan-targeted lymphatic endothelium to boost effector T cell immunity.

## Highlights

• Glycan under-sulfation boosts model antigen presentation by lymphatic endothelium.
• CD8+ T cell activation is augmented by antigen-pulsed glycan targeted lymphatics.
• This state drives increased lymph node CD8+ T cells with reduced metastases in vivo.
• Glycan targeting of lymphatic endothelium may augment anti-cancer adaptive immunity.

## Introduction

Lymphatic endothelium mediates key circulatory and immune physiologic roles. These include serving as a conduit for immunologic cell traffic and mediating critical cell-cell and microcirculatory roles in secondary and tertiary lymphoid organs [1,2]. In the carcinoma microenvironment, lymphatic trafficking of tumor cells to regional lymph nodes has major impact on prognosis and mortality [3]. Lymphatic tumor cell traffic to the draining lymph nodes (DLNs), which includes apoptotic tumor cells and free tumor antigen, provides an immunologic substrate for adaptive anti-tumor immunity in the DLN [4]. In carcinomas, this is challenged by immunologic exhaustion and a highly dynamic tumor antigen landscape in the heterogeneous tumor [[5], [6], [7]]. We sought to explore new biological insights on novel strategies to empower alternative antigen presenting cells in the tumor microenvironment (TME). We had a special interest in recruiting lymphatic endothelial cells (LECs) as non-myeloid cells to facilitate immune effector functions in carcinoma DLNs. In particular, the lymphatic endothelium contributes to a steady state of self-antigen flow and cross-presentation that promotes peripheral tolerance [[8], [9], [10]]. While these forces may contribute to self-antigen tolerance in tumors [11], expressing many self tumor-associated antigens into the DLNs, subsets of effector CD8+ T cells with long-term memory may also evolve by this process [9,10]. Methods to empower the micro-lymphatic conduit of DLNs to promote CD8+ T cell effector functions are especially intriguing when considering the lymphatic surface area and the kinetics of CD8+ T cells in the lymph node during early DLN-metastatic events: This is in the spirit of achieving statistically greater CD8+ T cell signal-1 contact events, even if the boost in antigen presenting capacity per LEC may be modest.

A small body of work has explored regulatory roles of cell-surface glycans on antigen-presenting dendritic immune cells (DCs) whereby altering or targeting glycosaminoglycans or sialic acids on the DC surface can modulate and even improve “signal 1″ (MHC-I/antigen presentation to the T cell receptor) in contexts of experimental neoplasia or viral infection [[12], [13], [14], [15]]. We here explored how introducing a specific glycan mutation in heparan sulfate (HS) biosynthesis into lymph node lymphatic endothelial cells might modify and possibly augment their capacity to activate antigen-sensitized CD8+ T cells. We were partly inspired by discovering in prior studies that genetic deficiency in the enzyme *N-deacetylase/N-sulfotransferase-1* (*Ndst1*) in DCs, associated with under-sulfated HS on the DC surface, was sufficient to increase tumor-associated CD8+ T cell influx and inhibition in tumor growth in mouse cancer modeling [12,16]. We also sought to test herein how antigen presenting capacity and CD8+ T cell activation might possibly be increased by *Ndst1*-deficient lymph node LECs in vitro. Despite a typically tolerance-driving setting of antigen in the context of MHC-I on lymphatic endothelium, along with minimal immune checkpoint molecule expression on the LEC surface [11,17], we tested the possibility that augmented antigen presentation and activation of sensitized CD8+ T cells might be achieved by LECs bearing under-sulfated cell-surface HS. From a practical standpoint and from whatever arbitrary tolerance or effector promoting “bias” of lymph node LECs in vitro or in tumor modeling, we investigate the relative effect of *Ndst1* glycan-targeting mutation in this unique and spatially abundant mesenchymal-derived cell with unique immunologic potential.

## Results

**Fig. 1:**
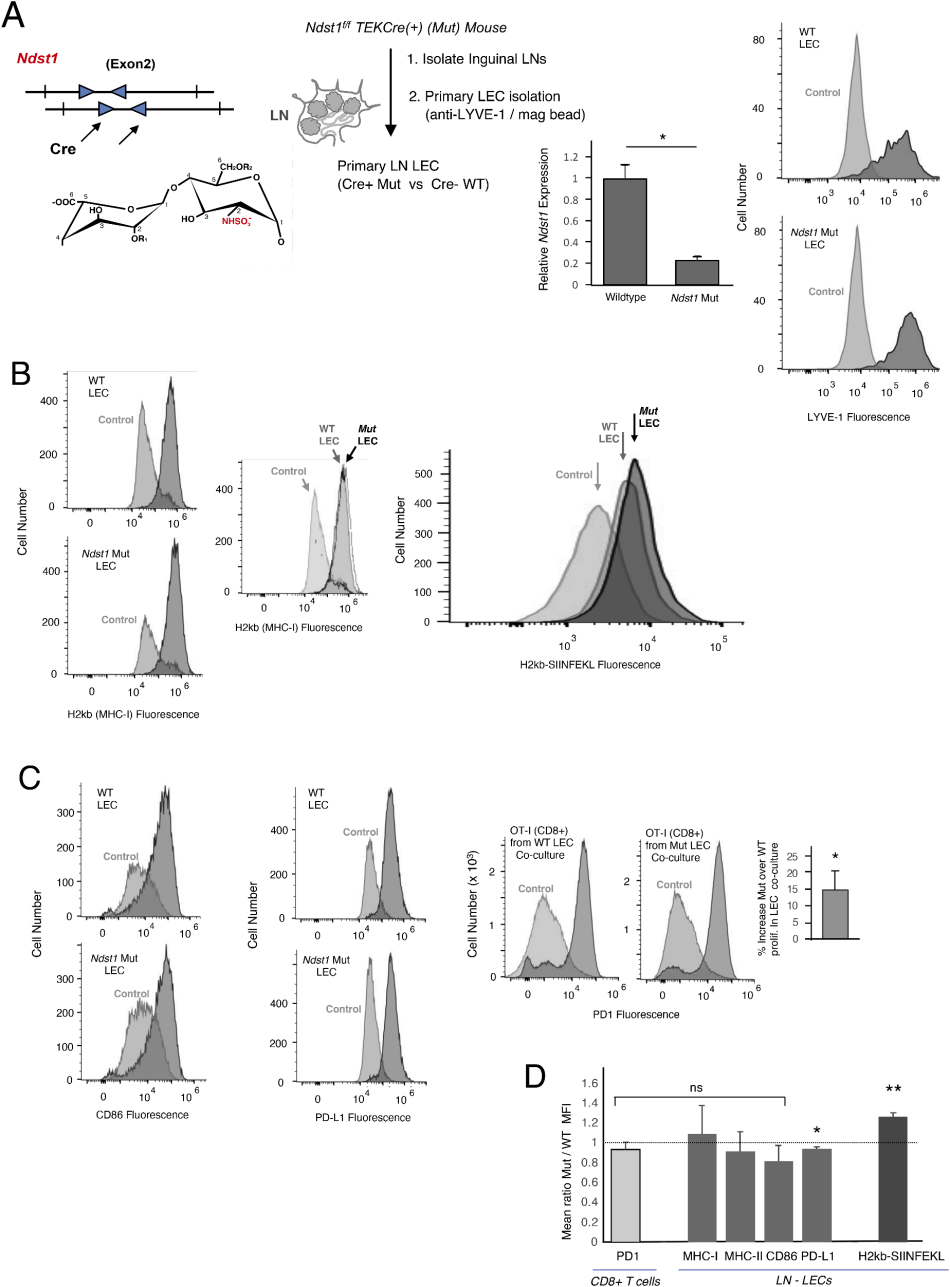
**Model antigen display and cell surface properties of lymph-node derived primary lymphatic endothelial cells with a mutation in a major sulfating enzyme involved in heparan sulfate biosynthesis.** Primary lymphatic endothelial cells (LECs) were isolated from the mediastinal lymph nodes of mice harboring a pan-endothelial mutation in the major HS sulfating enzyme *Ndst1* (**A**), responsible for N-sulfation of GlcNAc residues (shown in red) on nascent HS chains (where R_1_ or R_2_ may be replaced by -H or -SO_3_). Purified LECs from *Ndst1*f/f *Cre*+ mutant mice and *Cre*- wildtype littermates were confirmed as LYVE1+ by flow cytometry (far right), and showed significant reduction in expression *Ndst1* by qPCR (graph at right, normalized to wildtype control; P < 0.05 for difference in means). (**B**) LEC surface MHC-I as detected by anti-H-2 kb antibody by flow cytometry was not altered in mutant cells; however, following pulsing LECs with the Ova model antigen peptide SIINFEKL, binding of antibody that detects SIINFEKL bound to H-2 kb (clone 25-D1.16) demonstrated ∼ 40% increase in binding to mutant LECs relative compared to that of wildtype LECs (right). (**C**) In peptide-pulsed LEC co-cultured with OT-I transgenic CD8+ T cells (with T-cell receptor specific for SIINFEKL/H-2 kb) for 48 hr, expression of CD86 and PD-L1 checkpoint markers was similar among genotypes (left histograms) and PD-1 expression on co-cultured OT-I T cells was also similar (right). Nevertheless, expansion of OT-I cells by the mutant LEC was significantly greater than that by wildtype LEC (∼ 15% increased; graph far-right; P < 0.05 one-sample T-test). (**D**) Graph summarizes data for multiple flow cytometry trials carried out for each entity in (B) and (C): Each histogram bar depicts mean values for mutant (-fold MFI/control) over that for wildtype for multiple flow cytometry experiments (n = 4 experiments for MHC-I (H2kb), and n = 3 experiments for each of PD1, MHC-II, CD86, PD-L1, and H2kb-SIINFEKL conditions). Mean values for mutant expression relative to wildtype were significantly elevated for antigen expression on MHC-I (H2kb-SIINFEKL; 27% increase) and slightly reduced (∼6%) for PD-L1, while not significantly different from 1.0 for all other conditions (“ns” on graph). *P < 0.05 for significance of mean mutant value over that of wildtype (one sample T-test). (MFI = mean fluorescence intensity).

**A lymphatic endothelial mutation in** ***Ndst1*** **increases model antigen display on MHC-I along with a modest boost in the capacity to stimulate proliferation of co-cultured antigen-cognate CD8+ T cells.** Primary lymphatic endothelial cells (LECs) were isolated from the lymph nodes of *Ndst1*f/f *TekCre*+ mice that harbor a pan-endothelial mutation in the major HS sulfating enzyme *Ndst1* (Fig. 1A). This enzyme is important in N-sulfation of GlcNAc in HS disaccharide units (shown in red) in clustered regions of nascent HS polymers during HS biosynthesis; typically driving 2-O- and 6-O-sulfation on the same disaccharides [18]. Lymph node LECs from *Cre*+ mutant and *Cre*- wildtype littermates were purified, with Lyve1+ staining by flow cytometry and *Ndst1* deletion efficiency confirmed in the cultured primary LECs (Fig. 1A, right). While mutant and wildtype cells demonstrated no differences in the amount of cell-surface MHC-I by flow cytometry, mutant LECs had an increased capacity to present model SIINFEKL Ova antigen in the context of MHC-I following short antigen pulsing in culture, without significant differences in MHC-I expression (Fig. 1B, right; Fig. 1D). Otherwise, in the same experiments examining peptide-pulsed LEC co-cultured with OT-I transgenic CD8+ T cells (with T-cell receptor (TcR) that specifically recognizes SIINFEKL/H2kb) for 48 hr, expression of co-stimulatory (CD86) and co-inhibitory (PD-L1) ligands was detected in both mutant and control LECs. While there were no major differences in mutant versus wildtype LECs in expression of these ligands, expression of PD-L1 by mutant LECs showed a slight but significant reduction compared to that of wildtype LEC (Fig. 1C, left and Fig. 1D). In addition, the presence of mutant LEC in co-culture with OT-I T cells resulted in a modest but significantly greater expansion of OT-I cells relative to that of co-culture with wildtype mutant LEC (Fig. 1C, far right). Otherwise, the LEC genotype did not result in significant differences in PD-1 expression by the co-cultured OT-I T cells (Fig. 1C, and Fig. 1D, left).

**Fig. 2:**
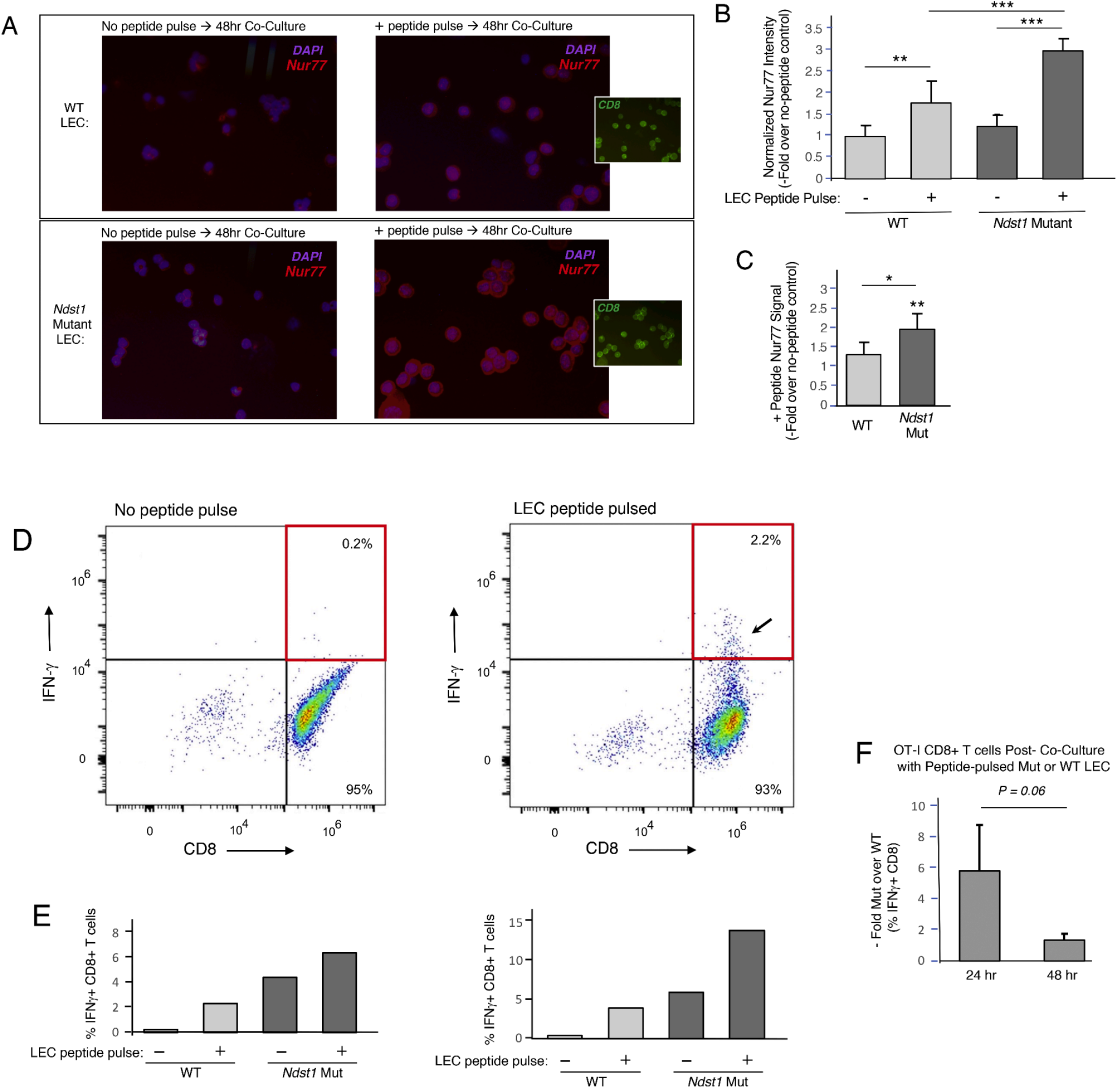
**Model-antigen pulsed LECs genetically deficient in*****Ndst1*****drive augmented Nur77 and IFN-g responses by co-cultured antigen-responsive CD8+ T cells.** Mutant lymph node LECs deficient in a major sulfating enzyme (*Ndst1*) involved in heparan sulfate biosynthesis and wildtype control LECs were pulsed for 2 hr with the Ova OT-I peptide SIINFEKL, and co-cultured for 48 hr with antigen-responsive OT-I transgenic CD8+ T cells that recognize SIINFEKL in the context of MHC-I. (**A**) Cytospin preparations of CD8+ T cells collected from the LEC co-culture layers at 48 hr were imaged for Nur77 immunofluorescence (red signal; with DAPI showing nuclei in purple), with representative photomicrographs shown at top for wildtype LEC co-culture pre-pulsed in the presence (right panel) or absence (left panel) of antigen peptide. The inset far-right image confirms CD8 expression by the T cells in the green-fluorescent channel. Bottom panels show Nur77 CD8+ T cell responses after co-culture with ± peptide-pulsed *Ndst1* mutant LECs. (**B**) Mean Nur77 expression responses (cell red-signal density) were quantified from n = 9 independent high-power (40X) cytospin fields; with responses normalized to the mean signal for wildtype no-peptide control. (**P < 0.01 for difference in means; ***P < 0.001 for difference). (**C**) Four independent experiments as in (B) were carried out, and for each experiment, the ratio of Nur77 signal in CD8+ T cells co-cultured with peptide-pulsed over that of non-pulsed cells was calculated for mutant LECs as the antigen presenting cell versus that of wildtype LECs. The means for the four experiments are shown in the graph; with –Fold peptide-specific CD8+ T cell Nur77 signal over no-peptide control shown for wildtype LECs (left bar) and *Ndst1* mutant LECs (right bar). (**P < 0.01 for significant difference from a ratio of 1.0 among 4 experiments; one-sample T-test; *P = 0.05 for difference in means for mutant versus wildtype). In similar experiments: (**D**) Co-culture for 24 hr after peptide-pulsing of wildtype LECs results in a subset of the CD8+ T cells harvested from co-culture demonstrating marked expression of the activation/ cytolytic cytokine IFNg (right panel, double-positive cells) relative to that of CD8+ T cells harvested from co-culture with non-pulsed LECs (left panel). Graphs in (**E**) show quantified values for IFNg+ CD8+ T cells (as % total co-cultured CD8+ T cells) from two representative 24 hr co-culture experiments demonstrating augmented capacity for peptide-pulsed *Ndst1* mutant LECs to generate IFNg/CD8 double positive cells (right, dark-grey bars) compared that of peptide pulsed wildtype LECs (left, light-grey bars). (**F**) Data from n = 3 experiments carried out over 24 hr of co-culture (left bar) or n = 4 experiments carried out over 48 hr of co-culture (right bar) shows the -fold (ratio) of IFNg/CD8 double positive cells harvested from co-culture with mutant LECs over that of wildtype LECs (P < 0.06 for difference in means).

**Antigen-pulsed lymphatic endothelial cells with under-sulfated HS drive augmented Nur77 and IFN-g responses by co-cultured antigen-responsive CD8+ T cells.** The findings above suggest increased antigen presentation by *Ndst1* mutant LEC over that of wildtype cells, following incubation with free peptide. We investigated how this mutation that results in under-sulfated LEC cell-surface HS might affect activation of co-cultured OT-I CD8+ T cells with a transgenic T cell receptor (TcR) that recognizes the OT-I peptide (SIINFEKL). Strikingly, following SIINFEKL peptide pulsing of LECs, and 48 hr co-culture with OT-I T cells, co-culture with *Ndst1* mutant LECs resulted in a marked increase in immunofluorescence-detected Nur77 signal in co-cultured OT-I T cells (red cytoplasmic signal in Fig. 2A). This TcR early-response transcription factor activates in CD8+ T cells upon antigen recognition by antigen presenting cells, and serves as a specific reporter of early antigen recognition and activation by cognate CD8+ T cells [19,20]. Mean OT-I CD8+ T cell Nur77 immunofluorescence intensity after 48 hr co-culture was increased in the setting of peptide-pulsed wildtype LECs compared to that of co-culture with non-pulsed controls (quantified in left bars of graph in Fig. 2B). The OT-I T-cell Nur-77 boost by co-cultured peptide-pulsed mutant LECs (Fig. 2B, right bars) was markedly elevated compared to that of wildtype LECs. In carrying out 3 independent experiments (as in Fig. 2B), the peptide-specific response by OT-I T cells co-cultured with mutant LECs was nearly double that of control cells (Fig. 2C).

In separate experiments, co-culture for 24 hr after peptide-pulsing of wildtype LECs resulted in a modest subset of the co-culture harvested CD8+ T cells demonstrating marked expression of the activation cytokine IFN-γ relative to that of CD8+ T cells harvested from co-culture with non-pulsed LECs (Fig. 2D). Quantified values for IFN-γ+ CD8+ T cells (as % total co-cultured CD8+ T cells) from representative 24 hr co-culture experiments (graphed in Fig. 2E) demonstrate an augmented capacity for peptide-pulsed *Ndst1* mutant LECs to generate IFN-γ/CD8 double positive cells compared to that of peptide pulsed wildtype LECs. Quantifying data from multiple experiments carried out over either 24 hr or 48 hr of co-culture further demonstrated that *Ndst1* mutant LECs drove greater induction of IFN-γ+ CD8+ T cells relative to that of wildtype LECs (summarized as -fold mutant/wildtype values in Fig. 2F) at the early (24 hr) period with attenuation in this effect over time, albeit still with slightly greater ratio of mutant over that of wildtype after 48 hr of co-culture.

**Fig. 3:**
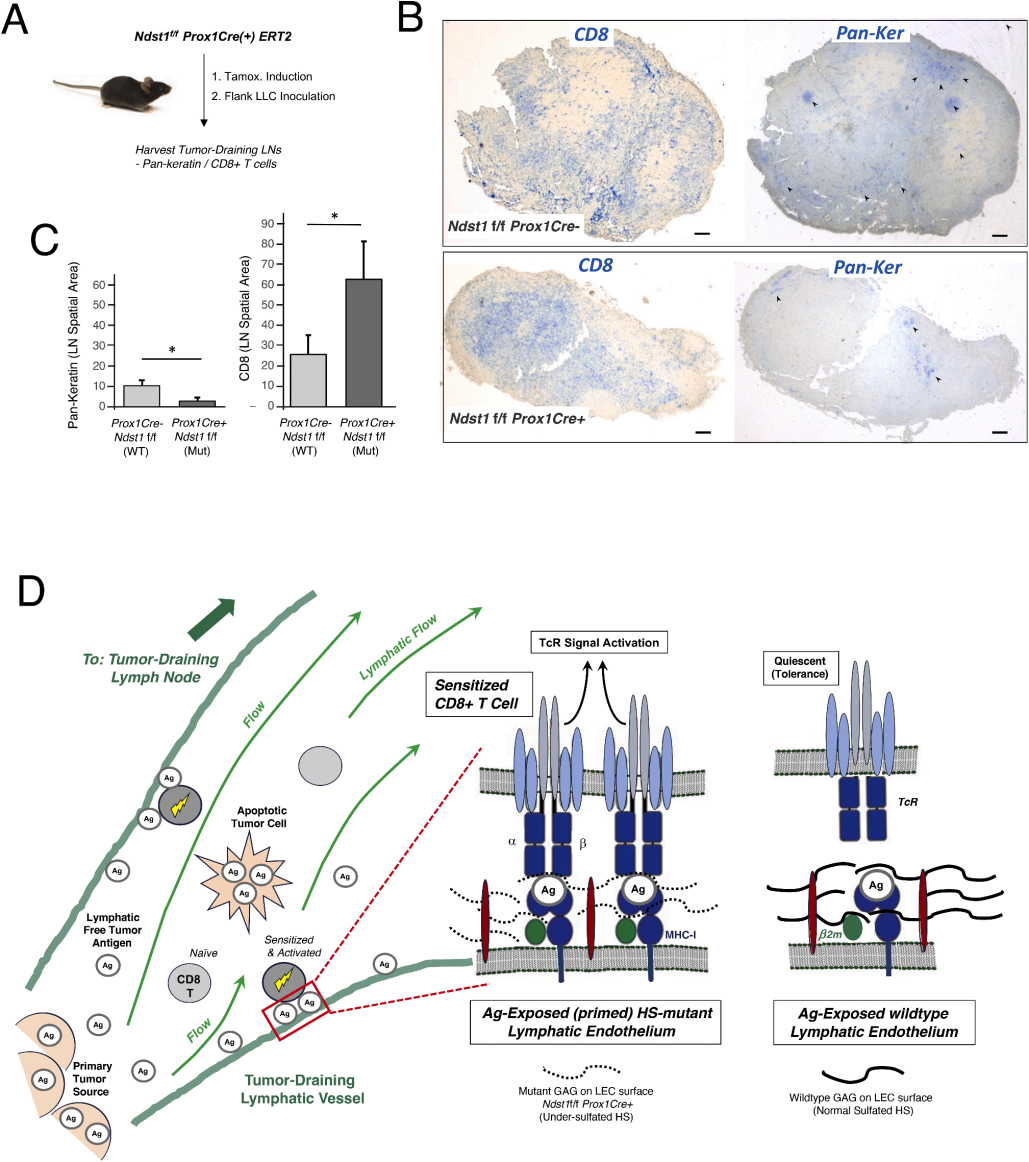
**Under-sulfated glycan expression in the tumor lymphatic microenvironment augments the anti-tumor CD8+ T cell response in tumor-draining lymph nodes. (A)** Lewis lung carcinoma (LLC) inoculation was carried out (subcutaneous tumor in the hindquarter) in *Ndst1*f/f *Prox1Cre*+ ERT2 mutant mice after tamoxifen induction to drive *Ndst1* deficiency and thus HS under-sulfation in lymphatic endothelium. Tumor-draining inguinal lymph nodes were harvested after a 2-week primary-tumor growth period, and immunohistochemical staining was carried out for CD8+ T cells as well as pan-keratin to identify tumor deposits within the lymph node parenchyma, with representative examples shown in **(B).** Confluent areas of dense CD8+ T cell expansion filled most the lymph node area in lymph nodes from mutant mice, while pan-keratin staining in the same mutant lymph nodes showed minimal tumor involvement compared to that of wildtype nodes (arrows in pan-keratin LN sections pointing to blue-stained tumor deposits). **(C)** Values and differences among *Prox1Cre*- wildtype mice versus that of *Prox1Cre*+ mutants were quantified by scoring of whole LN sections for spatial % LN area covered by either CD8+ T cells or pan-keratin by a staff pathologist blinded to genotype. Mean values are shown in the graph for n = 4 mice per genotype for CD8+ T cells (left bars) and for n = 4/ n = 3 mice for pan-keratin staining (right bars) (*P < 0.05 for differences in means; For photomicrographs: Bar = 100 mm). **(D)** Schematic showing lymphatic microenvironment draining both tumor cells (including apoptotic antigen (Ag) loaded cells) as well as free Ag with lymph flow toward draining LN afferent lymphatics in green. This may also represent interactions within lymphatics of the draining LN. While interactions with lymphatic endothelial cells (LEC) may drive tolerance in the wildtype setting, mutant LEC with under-sulfated HS (illustrated here) may form augmented interactions with effector Ag-sensitized CD8+ T cells (inducing activation and anti-tumor responses), highlighted by red-box region expanded to right: This demonstrates under-sulfated HS (dotted HS chains attached to red HS core proteins on the LEC surface) and clustering of signal-1 interactions with the TcR of sensitized CD8+ T cells, and activation. Whether this de-regulates beta-2 macroglobulin (b2m) on MHC-I to interact with other MHC-I subunits is unknown, as b2m is known to bind to wildtype/sulfated HS. This mutant scenario is to be contrasted with quiescent (or even tolerance-driving) state (right) showing an absent TcR interaction with Ag/MHC-I on wildtype LECs.

**Lymphatic endothelial HS under-sulfation is sufficient to augment anti-tumor CD8+ T cell responses in Lewis lung carcinoma tumor-draining lymph nodes.**In mice carrying a conditional mutation in *Ndst1* (inducible via tamoxifen induction) targeted to the lymphatic circulation under control of *Prox1Cre* (Fig. 3A), we examined tumor draining lymph nodes (DLN) at 2 weeks following tumor inoculation in subcutaneous growth models. Immunohistochemical staining was carried out for CD8+ T cells and pan-keratin to identify tumor deposits within the lymph node parenchyma, and we noted a pattern of increased CD8+ T cell density and coverage across the lymph node that correlated with reduced pan-keratin staining in the DLNs of *Cre*+ mice with mutant LECs (with photomicrographs and graphs in Fig. 3B,C). While the graph shows significant differences in DLN CD8+ T cell and metastatic tumor cell spatial densities as they relate to genotype, as determined on slide-review by a staff Pathologist (blinded to genotype), a direct calculation of ratios of CD8+ T cell / pan-keratin density for each mouse also resulted in a higher ratio of effector T cells to tumor cells in mutant versus wildtype mice (*P* = 0.058 for difference in means). Fig. 3D shows a schematic illustration of the lymphatic TME with a focus on the tumor DLN, wherein lymph flow carries antigen into DLNs while resident lymphatic vessels in the DLN may also encounter apoptotic tumor cells or even free tumor antigen. A mutant lymphatic endothelium is depicted, wherein augmented events of tumor antigen presentation on MHC-I of LECs (that line the lymphatics, in green) is shown; and inset in the figure showing how under-sulfation of HS proteoglycans (dotted chains on surface of the LEC) surrounding MHC-I molecules may facilitate MHC-loaded antigen stability/ clustering, and ultimate stimulation of primed CD8+ T cell activation. In wildtype DLNs, shown to far right, where native HS is more sulfated, tumor antigen presentation is not augmented to the degree of the *Ndst1* mutant under-sulfated HS state. Whether mutant HS on the LEC surface somehow de-regulates beta-2 macroglobulin (β2 m) on MHC-I to interact with other MHC-I subunits is unknown, as β2 m is known to bind to wildtype/sulfated HS.

## Discussion

We show herein that targeting a *Ndst1* mutation to lymphatic endothelium, leading to under-sulfated lymphatic vascular heparan sulfate, results in augmented early CD8+ T cell activation in response to model antigen presentation. This follows co-culture of transgenic antigen-specific OT-I CD8+ T cells with antigen-peptide pulsed mutant LECs, while the in vivo LEC mutation appears to augment the density and possibly function of CD8+ T cells in tumor draining LNs while inhibiting DLN metastatic tumor growth. This finding in the setting of tumor modeling brings insight into novel potentially inducible biological states wherein a typically tolerogenic lymphatic endothelium in cancer might be harnessed to “tilt an immunologic balance" toward driving effector/activating responses with antigen-cognate CD8+ T cells in the same tumor microenvironment (TME). This in turn may induce augmented anti-tumor immunity in the lymphatic and DLN microenvironment while LECs, even as mesenchymal derived cells, may serve in expanding anti-tumor CD8+ T cell immunity to locally released cancer antigenic peptides.

While LECs serve circulatory functions and support immunologic cell trafficking through their integration with secondary and tertiary lymphoid organs, their ability to serve as antigen presenting cells (APCs) for foreign/ neo-antigens appears to be limited [9,11]. As mesenchymal cells, while able to present antigen on MHC-I, lymphatic endothelium is known to lack co-stimulatory molecules. The presence of PD-L1 on the LEC surface with a paucity of immune synapse ligands typically confers a tolerance-promoting regulatory function [17] for this TME cell. Inspired by our prior observations that cell-surface HS under-sulfation facilitates antigen presentation by dendritic immune cells as “professional” APCs that serve key but spatially restricted roles in TME tumor antigen presentation, we sought to assess whether inducing HS under-sulfation via *Ndst1* mutation in LECs may possibly “tip” or bias lymph node LECs toward more effective antigen-presenting functions. This might also allow CD8+ T cells in the TME to activate in response to a broader display of tumor antigens over the very conduit through which the T cells traffic.

We focused initial in vitro studies uniquely on LECs purified from the lymph nodes of *Cre-LoxP* targeted animals with a pan-endothelial mutation in *Ndst1* (Fig. 1A) Curiously, purified *Ndst1* mutant LECs showed modest increases in model-antigen presentation on MHC-I compared to that of wildtype/control LECs without notable increases in total MHC-I (mouse H2k-b) on mutant LECs (Fig. 1B). This was reminiscent of a similar functional discovery we made in prior studies in DCs genetically deficient in the same glycan biosynthesis enzyme [12]. While two key immune checkpoint molecules CD86 (activating) and PD-L1 (inhibitory) were detected on the surface of such LECs, mutation did not result in marked differences in expression of these molecules (save for a slight but significant reduction in PD-L1 on mutant LECs; Fig. 1D) or in PD-1 on model-antigen responsive transgenic CD8+ T cells that were co-cultured with mutant versus wildtype LECs (Fig. 1C, D). Strikingly, the capacity to activate Ova (OT-I peptide) antigen cognate OT-I transgenic CD8+ T cells by peptide-pulsed LECs was markedly increased in *Ndst1* mutant LECs as compared to that of wildtype controls (Fig. 2A,B), making us consider whether immunologic “signal 1″ (i.e., MHC-I/antigen – TcR interactions) is augmented by the mutation, reminiscent of glycan-targeted DCs in this setting. Curiously, these activation responses were somewhat out of proportion to a more modest but still significant boost in proliferation of T cells co-cultured with mutant LECs over that of wildtype (Fig. 1C, far right). Moreover, while more modest in magnitude, early IFN-γ responses to antigen-pulsed LECs by antigen-responsive transgenic CD8+ T cells were also augmented relative to that of wildtype LECs (summarized quantitatively in Fig. 2D-F).

These observations prompted us to investigate whether augmented CD8+ T cell responses are present and correlate in any way with reduced tumor metastasis/proliferation in the DLNs of model carcinoma studies in mice bearing an inducible form of the same under-sulfating glycan-biosynthesis mutation targeted specifically to LECs in vivo. In *Ndst1*f/f *Prox1Cre* ERT2 mutant mice (Fig. 3A, model), following tamoxifen induction of lymphatic-directed *Ndst1* deficiency and tumor inoculation/ growth of LLC tumors, we noted reduced pan-keratin in the inguinal DLNs of *Cre*+ mutant mice (Fig. 3B and C, left bars), highlighting reduced DLN epithelial/tumor cells compared to that of *Cre*- wildtype controls. While this recapitulated similar findings in prior work with this strain [21], we additionally quantified CD8+ T cells in the same nodes (Fig. 3B and C, right bars). We found that significant increases in CD8+ T cell density in the DLNs of *Cre*+ mutants were associated with tumor inhibition.

Our findings suggest that possibly, even a modest bias toward effector-driven antigen presentation on glycan-targeted peri‑tumoral LECs may lead to marked effects on primed trafficking T cells. The surface area of the LN lymphatic microvasculature may serve as a relatively large “antigen-presenting surface” for pre-metastatic tumor cells that have evolved their antigen landscape, with LN tumor metastases as prime targets for augmented adaptive anti-tumor immunity in the setting of carcinoma. In the primary tumor and DLN, a minimal capacity for LECs to cross-present neo-antigens to induce long-lasting memory T cell responses (as reported in [10]) may be paralleled by the ability to bind substantial tumor antigen directly on lymphatic MHC-I. While often involved in tolerance induction under steady state of non-tumor self-antigens, LECs exposed to sufficient exogenous tumor “pulsed antigen” accompanied by cytokines that up-regulate vascular MHC-I expression [22,23] in the TME may create conditions to drive activation of trafficking antigen-sensitized CD8+ T cells. Glycan targeting through lymphatic HS under-sulfation, as demonstrated herein, may thus potentiate recognition of the tumor-antigen “pulsed” surface of DLN afferent lymphatic vessels by trafficking CD8+ T cells. In the setting a sufficient pathogen or tumor load, antigen presentation of lymphatic MHC-I in this setting may be sufficient to activate sensitized CD8+ T cells [24]. The magnitude of this process may lie in the amount of vascular (in this case lymphatic) surface area potentially available in the lymphoid microenvironment of the tumor DLNs.

One may also postulate that under-sulfating HS, as a key binding molecule for CCL21 in peri‑lymphatic matrix and the LEC surface, may release bound CCL21 pools to attract CCR7 responsive CD8+ T cells toward LN sites of augmented CCL21 production over longer distances [25]. This may promote infiltration of tumor-colonized LNs by CD8+ T cells, as has been demonstrated in engineered approaches to augment CCL21 in the TME to boost anti-tumor immunity in vivo [26]. Pairing this with strategies to boost the release of tumor associated immunodominant peptides in the TME, including approaches to drive tumor apoptosis in parallel to increased antigen association with MHC-I [27] may synergize with such novel strategies to augment CD8+ T cell presence.

We corroborate the above in vivo findings with model-antigen activation responses in Fig. 2 along with the possibility of augmented antigen presentation (Fig. 1B) to primed CD8+ T cells in this novel lymphatic microenvironment. In vivo, we cannot not rule out the possibility of a reduced rate of metastasis (i.e., tumor-cell *trafficking*) to the DLN that may result from the lymphatic *Ndst1* mutation; however, the collective findings herein point to the new insight that afferent and DLN LECs with under-sulfated HS are able to augment CD8+ T cell responses in the lymphatic microenvironment *in situ* (summarized in Fig. 3D). Regardless of the rates of incident metastatic cells to the DLN from the primary tumor among mutant and wildtype states, the possibility that the growth of a metastatic “seed” is more inhibited in mutant DLNs remains plausible in the setting of greater DLN CD8+ T cell abundance in the same nodes (Fig. 3B,C). It is also possible that LN-resident DCs and/or macrophages may secondarily activate and/or expand in this setting, with potentially secondary antigen transfer events (e.g., to follicular dendritic cells or other resident APCs [28]). These cells may also present antigen arriving via afferent LN lymphatic vasculature, which may further contribute to cytotoxic T-cell killing of incident lymphatic tumor metastases. Finally, it is intriguing from a translational standpoint to consider the possibility that concomitant blockade of the PD-L1/PD-1 axis in the setting of under-sulfated HS on LECs may lead to yet greater CD8+ T cell activation given PD-L1 presence on both tumor cells and on LECs [1,17] that may be additionally signal-1 “primed” in the *Ndst1* deficient setting.

## Conclusion

A glycan mutation in LECs appears to serve as a novel way to boost adaptive immunologic behavior by this mesenchymal-derived cell that is spatially critical and abundant in afferent DLN lymphatics and possibly tumor-surface/ draining lymphatics in primary carcinomas. Using genetically targeted LECs in vitro and in vivo, we demonstrate augmented antigen presentation upon exposure of MHCI immunodominant peptide and CD8+ T cell activation in the unique state of genetically under-sulfated LEC glycosaminoglycan. With an eye to possibly “recruiting” the TME to augment anti-tumor adaptive immunity, the biological concept of expanding the spatial area over which such events take place is intriguing; and with nano-targeting of small-molecule glycan biosynthesis inhibitors to the lymphatic TME, such strategies may be appealing from a translational standpoint. More generally, the possibility that antigen presentation to sensitized CD8+ T cells in the lymphatic microenvironment might serve as a way to expand responses to foreign antigens, including contexts of viral or other microbial infection for example remains plausible, but our focus in preliminary insights herein was driven by the carcinoma setting.

## Resource Availability

Mouse lines described, including *Ndst1*f/f mutant mice, may be provided upon written request, and under appropriate materials transfer agreement. We are happy to make available other reagents such as OT1 T cells, primers, antibody reagents, and/or other immunologic reagents.

## Declaration of Interests or Generative AI

The authors declare no financial or other interests in relation to this work. There has been no use of generative AI in the creation of this work.

## CRediT authorship contribution statement

**Aditya Dash:** Data curation, Formal analysis, Investigation, Methodology, Writing – review & editing. **Scott C. Johns:** Data curation, Formal analysis, Investigation, Methodology, Resources, Supervision. **Chace Moleta:** Data curation, Formal analysis, Investigation. **Elina I. Zúñiga:** Resources, Validation, Writing – review & editing. **Mark M. Fuster:** Conceptualization, Data curation, Formal analysis, Funding acquisition, Investigation, Methodology, Project administration, Resources, Supervision, Validation, Writing – original draft, Writing – review & editing.

## Declaration of competing interest

None
